# Epipalaeolithic animal tending to Neolithic herding at Abu Hureyra, Syria (12,800–7,800 calBP): Deciphering dung spherulites

**DOI:** 10.1371/journal.pone.0272947

**Published:** 2022-09-14

**Authors:** Alexia Smith, Amy Oechsner, Peter Rowley-Conwy, Andrew M. T. Moore

**Affiliations:** 1 Department of Anthropology, University of Connecticut, Storrs, CT, United States of America; 2 Institut für Naturwissenschaftliche Archäologie, University of Tübingen, Tübingen, Germany; 3 Dept of Archaeology, Durham University, Durham, United Kingdom; 4 Rochester Institute of Technology, New Castle, NH, United States of America; University at Buffalo - The State University of New York, UNITED STATES

## Abstract

Excavations at Abu Hureyra, Syria, during the 1970s exposed a long sequence of occupation spanning the transition from hunting-and-gathering to agriculture. Dung spherulites preserved within curated flotation samples from Epipalaeolithic (ca. 13,300–11,400 calBP) and Neolithic (ca. 10,600–7,800 calBP) occupations are examined here alongside archaeological, archaeobotanical, and zooarchaeological data to consider animal management, fuel selection, and various uses of dung. Spherulites were present throughout the entire sequence in varying concentrations. Using a new method to quantify spherulites, exclusion criteria were developed to eliminate samples possibly contaminated with modern dung, strengthening observations of ancient human behavior. Darkened spherulites within an Epipalaeolithic 1B firepit (12,800–12,300 calBP) indicate burning between 500–700°C, documenting early use of dung fuel by hunter-gatherers as a supplement to wood, coeval with a dramatic shift to rectilinear architecture, increasing proportions of wild sheep and aurochsen, reduced emphasis on small game, and elevated dung concentrations immediately outside the 1B dwelling. Combined, these observations suggest that small numbers of live animals (possibly wild sheep) were tended on-site by Epipalaeolithic hunter-gatherers to supplement gazelle hunting, raising the question of whether early experiments in animal management emerged contemporaneously with, or pre-date, cultivation. Dung was used to prepare plaster floors during the Neolithic and continued to be burned as a supplemental fuel, indicating that spherulites were deposited via multiple human- and animal-related pathways. This has important implications for interpretations of archaeobotanical assemblages across the region. Spherulite concentrations dropped abruptly during Neolithic 2B (9,300–8,000 calBP) and 2C (8,000–7,800 calBP), when sheep/goat herding surpassed gazelle hunting, possibly corresponding with movement of animals away from the site as herd sizes increased. As hunter-gatherers at Abu Hureyra began interacting with wild taxa in different ways, they set in motion a remarkable transformation in the ways people interacted with animals, plants, and their environment.

## Introduction

**Fig 1:**
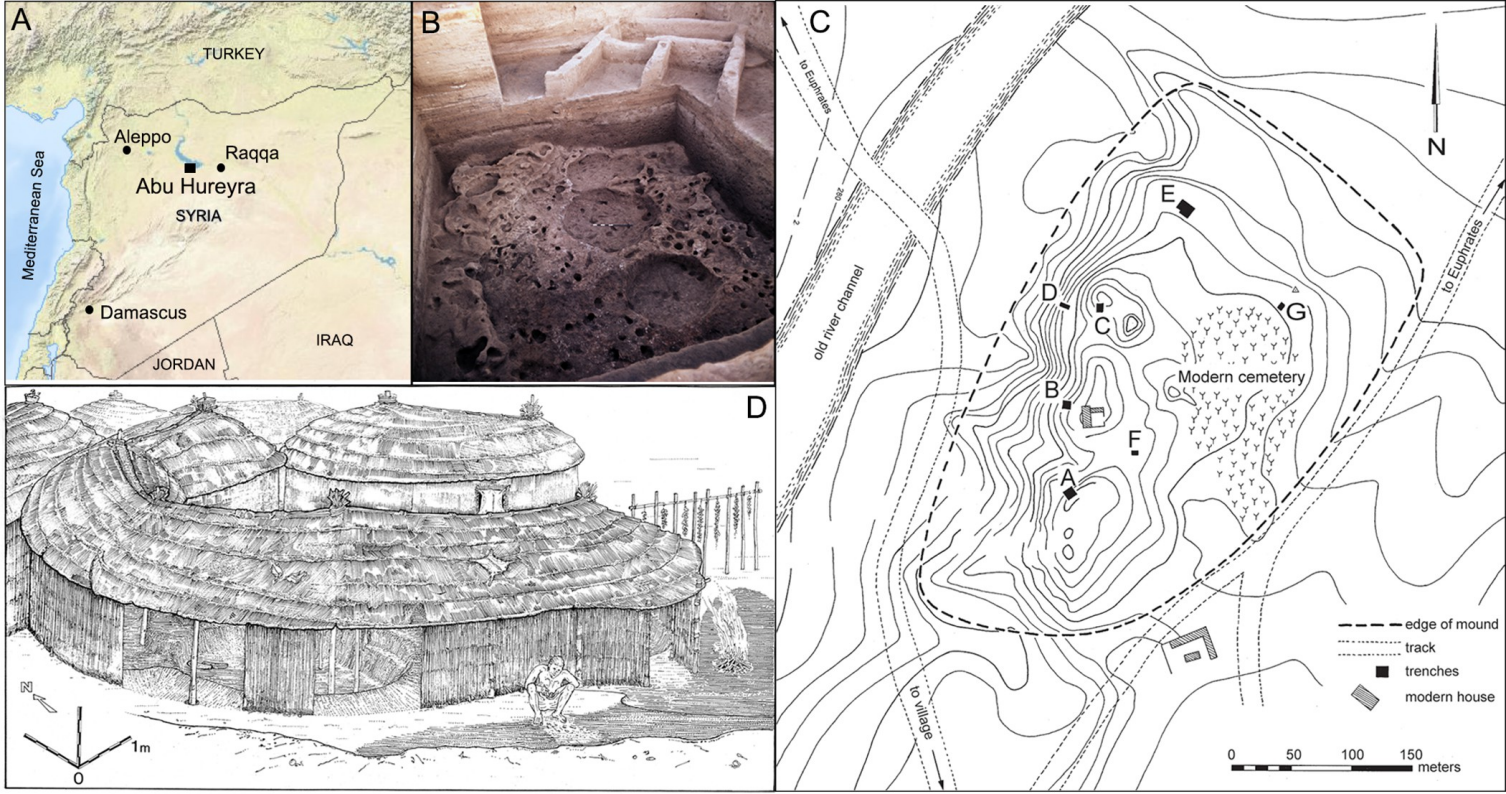
A) Map of Syria highlighting location of Abu Hureyra (base map obtained from USGS National Map Viewer, public domain); B) photograph of superimposed structures within Trench E showing Neolithic 2A rectilinear structure atop Epipalaeolithic 1A pit dwellings in the foreground; C) Plan of excavations at Abu Hureyra highlighting location of trenches A–G; and D) reconstruction of the phase 1A pit dwellings showing a person seated on the open “bench” area in the foreground.

Excavations at Abu Hureyra, Syria in 1972 and 1973 exposed one of the longest cultural sequences in Southwest Asia to document the transition from hunting-and-gathering to farming and herding, spanning the Epipalaeolithic (AH1, 1A–1C) and Neolithic (AH2: 2A–2C) periods [1–3] (Fig 1, Table 1). The site lay close to the Euphrates River, at the interface of an ecologically diverse floodplain to the north and fertile steppe and woodland–steppe to the south, providing ready access to waterways and abundant wild flora and fauna [1, 3–6]. As hunter-gatherers at Abu Hureyra and elsewhere began interacting with wild taxa in different ways, they set in motion a remarkable transformation in the ways people interacted with plants and animals and their environment.

**Table 1:** Chronology of occupation at Abu Hureyra. Approximate date ranges reflect radiocarbon dates calibrated using IntCal20 [1, 2].

Abu Hureyra phases	Date range (calBP)	Associated Period
1A	13,300–12,800	Epipalaeolithic
1B	12,800–12,300	Epipalaeolithic
1C	12,300–11,400	Epipalaeolithic
Intermediate	11,400–10,600	Early Neolithic
2A	10,600–9,300	Pre-Pottery Neolithic B
2B	9,300–8,000	Pre-Pottery Neolithic B
2C	8,000–7,800	Late Neolithic
3	Late Prehistoric and Historical periods	Ubaid to Present

Since the early contributions of scholars like Raphael Pumpelly [7] and V. Gordon Childe [8], numerous studies have contributed to discussions of the timing and location of domestication processes across the Fertile Crescent. Over the past several decades, our understanding of the transition from hunting-and-gathering to a large-scale reliance upon farming and herding has both broadened and deepened, with debate focusing on the details of a single core area or multiple locations for various taxa, the timing of a rapid or drawn out process of change, and the specific nature of various aspects of the transition [9–23]. During the 1990s, for example, work began focusing on early cereal domestication in the southern Levant during the Pre-Pottery Neolithic A (PPNA, ca. 11,500–11,000 calBP) [9]; by 2000, a single core area for multiple crops was proposed in the Karaca Dağ region of southeastern Turkey [10], with goat, sheep, and cattle domestication events thought to have occurred across the region later, during the Pre-Pottery Neolithic B (PPNB, ca. 10,000 calBP) [9, 11]. The general understanding remains that cultivation began first, tethering people to the land, and that herding followed. The research outlined here raises questions regarding this trajectory.

The growing realization that morphological markers of domestication on archaeobotanical and zooarchaeological remains emerge well after the beginnings of initial animal and plant management [11–14] has shifted the lens back in time. Numerous studies of plant [15–19] and animal [14, 20–22] remains have moved away from conceptual dichotomies, to consider the middle-ground between wild and domesticated, foraged and farmed, and hunted and herded, revealing a highly diffuse and pluralistic transition with tremendous geographic variation in the integration of “free-living, managed, and fully domesticated resources” [23].

While important insights have been gained regarding this elusive and incipient period, there is still a lack of clarity regarding the full range of human/animal interactions and practices that may have existed throughout the later Epipalaeolithic and early Neolithic, as dominant subsistence strategies shifted from hunting-and-gathering to herding and farming. This largely reflects a paucity of reliable markers with which to observe variable early behaviors archaeologically. The nature of human/animal interactions could have been highly diverse, fluid, and flexible, across both temporal and spatial gradients, and some of the practices may no longer exist [24]. Given this variation, it is no surprise that the existing lexicon struggles to encompass the potential changes and that different connotations have been applied to fundamental terms such as domestication, tending, management, and control [25, 26]. Indeed, these terms do not always relate to mutually exclusive behaviors, further complicating matters. The term “tending,” for example, implies a level of care or involvement that could range from short-term acts of feeding captured wild animals (which may or may not be behaviorally tamed), possibly as temporary “storage on the hoof” as others have argued [27], to more involved seasonal or year-round oversight that may involve periods of reproduction. Both involve close co-presence, but the nature of interaction clearly differs in intensity.

Recent studies of animal dung in Neolithic and post-Neolithic sites have proven successful in recording a range of human activities that involve plants and animals, including on-site animal tending, penning, and use of dung as a fuel or construction material [28–33]. Despite the enormous potential of dung studies to explore the presence of animals on-site, they have not yet been widely applied to Epipalaeolithic sites to examine any changing relationships between people and animals, to some degree reflecting the belief that no other practices outside of hunting-and-gathering could have existed at that time (an assumption that was lamented more than 50 years ago by Higgs and Jarman [34] in their seminal article on the origins of agriculture).

**Fig 2:**
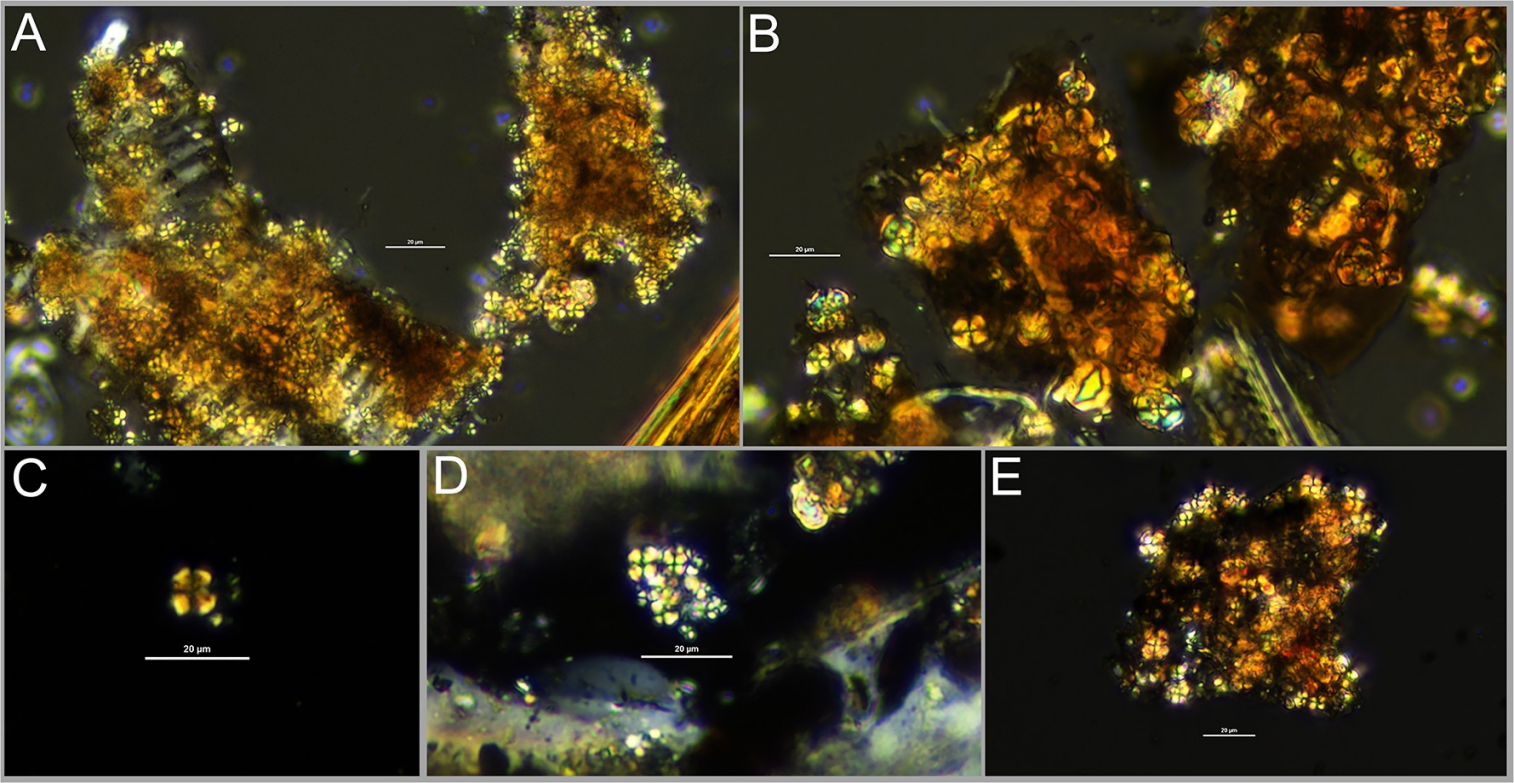
Photographs of spherulites within: A) modern uncharred gazelle (*Gazella gazella)* dung; B) modern uncharred goat (*Capra aegagrus hircus)* dung with organic “fibers”; and examples of C) an isolated individual spherulite; D) a 2-dimensional cluster of spherulites; and E) a 3-dimensional spherulite cluster. Scale bar in all images = 20 μm.

This study presents analyses of dung spherulites excreted by ruminant herbivores (Fig 2), within curated flotation samples from select archaeological contexts spanning the entire Epipalaeolithic and Neolithic stratigraphic sequence at Abu Hureyra, alongside published zooarchaeological and archaeological data, to examine the earliest presence of animals on-site, shifting animal management strategies through time, and potential uses for animal dung (including as a fuel and a construction material). As knowledge of early management strategies has deepened in the Zagros, southern Levant, and Central and Southeastern Anatolia, parallel developments in the middle Euphrates Valley have slowed, in large part owing to ongoing conflicts in the region. The use of well-curated legacy collections provides a means for continued study of the site and the wider region. This remains particularly true for Abu Hureyra, which now lies submerged beneath Lake Assad.

## Background

Various approaches have been used to track early control or management of food-based resources prior to the emergence of morphological markers of domestication [35]. Zeder and Hesse’s zooarchaeological study at Ganj Dareh, Iran, revealed management of goat herds around 9,900 calBP through selective culling of young-males and delayed slaughter of females [35, 36]. Incorporating lower resolution harvest data, they suggest that management of sheep and goat across the eastern Taurus and northwestern Zagros mountains could have begun as early as 11,000 years ago and anticipate that the “initial phases of the transition from hunting to herding in this region may…reach back to about 12,000 to 13,000 calendar years ago” [35]. In the southern Levant, Munro et al. employed behavioral ecology models to examine game management prior to selective culls, observing management of ungulate taxa in the PPNB (10,500–10,000 calBP) through combined consideration of relative taxonomic abundance and age data from both small and large wild game, and taxa that eventually became domesticated [14]. In particular, they note that relative decreases in small wild game associated with small increases in ungulate populations can be quite subtle, yet meaningful markers of an emerging relationship in the earliest stages of animal management. Similar management signatures were observed in faunal remains from the Southern Levant, Zagros, and at Aşıklı Höyük in Central Anatolia, where additional observations of dung accumulations between structures provide early evidence for on-site animal penning around 10,150 calBP [27, 37–39]. The introduction of morphologically wild animals via boat to Cyprus around 10,400 calBP, further suggests an established practice of control with earlier roots [12].

Observations of dung, when carefully considered alongside archaeological and zooarchaeological data, provide a means for exploring the temporal depth of these elusive behavioral roots and examining the co-presence of people and animals on archaeological sites. While the beginnings of these behaviors are thought to be largely embedded in the Neolithic, in 1998, Rosenberg et al. [40] argued for an even deeper reach at Hallan Çemi in southeastern Anatolia; survivorship and sex ratio data recorded from *Sus scrofa* (wild boar) remains, while morphologically wild [41], hint at mixed pig-rearing and pig hunting, including the capture and tending of young animals and a strong male culling bias during the terminal Epipalaeolithic. A similar practice of capturing wild lambs and kids for short term live meat storage was recently documented using multiple lines of evidence at the earliest Neolithic Level 5 at Aşıklı Höyük, dating to around 10,400 years ago [42].

Archaeobotanists have similarly focused on intensifying management of morphologically wild taxa, or pre-domestication cultivation, through combined consideration of seed and chaff morphology, shifts in wild/weedy seed assemblages and, more recently, genetic data [18, 43–46]. Hillman’s formative study of the Epipalaeolithic plant sequence from Abu Hureyra (AH1) has remained tremendously influential to ongoing conversations of plant domestication across Southwest Asia [19, 46–49]. The diverse plant taxa recovered have many ethnographic examples of use by both people and animals, and share similarities with remains from the earlier site of Ohalo II, Israel (23,000 calBP), where exceptional preservation of organic remains within huts yielded more than 142 plant taxa and abundant small-seeded grasses, linked to a broad-spectrum diet with a heavy emphasis on gazelle hunting alongside other game [50, 51].

In addition to food, plants are widely used for structures, furniture, tools, medicinal/psychoactive purposes, dyes, textiles, basketry, fodder or pasture, and fuel [1]. Each of these uses is associated with different preservation potentials, depositional pathways, and interpretive issues. The majority of macro-botanical remains from Southwest Asia are preserved via charring, and the vagaries of preservation favor taxa that are routinely exposed to fire and can withstand heat [52]. Studies of fuels (which have a high preservation potential owing to their link with fire), have historically fallen within the realm of anthracology, but phytolith and geoarchaeological studies are now contributing interesting perspectives. Observations that integrate macro-botanical data with other studies have much to offer to conversations of the socially-conditioned nature of fuel use [53–55] and considerations of sample deposition processes that help inform the range of questions that archaeobotanical data can be used to address.

In 1984, building upon ethnographic observations and archaeobotanical analyses at Bronze Age Malyan, Iran, Miller argued that spent dung fuel may contribute to archaeobotanical assemblages across Southwest Asia where dung-producing animals were present and the archaeological context was associated with fuel [56, 57]. Her work is relevant here because she later challenged Hillman’s interpretation of the Epipalaeolithic plant assemblage from Abu Hureyra [4], asserting that the remains reflected seeds excreted in dung that was burned as a fuel (as opposed to being remnants of food collected directly by people), thereby providing insights into animal rather than human diet [58]. Gazelle was viewed as the most likely contributor owing to their dominance in the faunal record, combined with the tendency of male gazelles to mark territory with dung piles that could be gathered by people for burning. While acknowledging the importance and possibility of burned dung as a depositional pathway for plants in later time periods, Hillman, Legge, and Rowley-Conwy rejected Miller’s argument for gazelle dung burning at Epipalaeolithic Abu Hureyra, maintaining the seeds had been intentionally collected for human consumption based on seasonal discrepancies between select plant taxa and the timing of mass gazelle hunting, the likely small and sporadic nature of dung piles, the perceived inability of gazelle to consume some of the taxa present, and the abundance of wood in many of the occupational deposits associated with fuel burning [1, 59]. Since their debate, which occurred in the late 1990s, diverging schools of thought on primary pathways of plant deposition across Southwest Asia have persisted, shaping questions asked of both macro-botanical and phytolith remains and influencing subsequent interpretations of the data generated [48, 60]. Given the universal need for food and fuel, it is reasonable to expect that both food- and fuel-related activities would have contributed to sediments in varying degrees across space and time, resulting in a range of food-derived, fuel-derived, and mixed assemblages representing a range of activities [30, 61]. It remains unclear, however, when the use of dung as a fuel began. This question is explored here. Elevated considerations of fuel use on par with questions of subsistence, combined with observations of the presence of dung on sites, allow for a deeper consideration of the relative contributions of each plant depositional pathway on a sample-by-sample basis, placing our understanding of ancient plant use on a firmer footing. Such approaches also provide a means for bridging the gap between studies of ancient plant- and animal-based economies [30, 53].

Archaeological dung can be studied in a variety of forms, including dung spherulites [62]. Dung spherulites are roughly 5–20 μm radially crystallized calcium carbonate bodies that form in the intestines of animals and, while a range of age, sex, diet, nutrition, health, and soil-related factors can affect production, they tend to be most abundant in ruminant herbivores (such as gazelle, sheep, goat and cow), low in omnivores (such as pig, dog, and humans), and are generally thought to be rare to absent in carnivores and caecal digesters such as horse [63]. They have been observed in mouflon, roe deer, chamois, and wild boar feces [64, 65]. Within our lab, we have also observed spherulites in hare pellets.

Dung spherulites exhibit a clear extinction cross surrounded by bands of low-order white, first order red, and second order blue under cross-polarized light [63] (Fig 2) and are distinguishable from starch grains through combined consideration of their size, color, and stable cross when the microscope stage is rotated [66]. Newly-discovered starch spherulites present possible confuser-types, but differ in size and visual characteristics [66, 67]. Spherulites do not require charring to preserve and can withstand high temperatures frequently encountered in hearths, although they do begin to break down when a certain temperature threshold is met [68]. Brochier [65] cites breakdown between 500–560°C while Shahack-Gross [62] cites 650–700°C. Based on our own observations, breakdown does not occur uniformly and instantly once a particular threshold is met, supporting this discrepancy; it is possible that the different temperature ranges reported in the literature relate to variable oxidizing conditions among other factors. Between 500 and 700°C, small proportions (ca. 0.2–25%) of spherulites become darkened, serving as an indicator of burning [69, 70], enabling identification of fuel use within pyric features. Spherulites dissolve within acidic conditions and sediments with high water flow-through, yet frequently occur on archaeological sites across Southwest Asia, where they have successfully documented animal penning, construction practices, and dung fuel use during the Neolithic [27, 32, 33, 70–72]. Micromorphological samples have been used with much success to examine the in situ micro-archaeological context of spherulites, allowing for detailed reconstructions of depositional sequences [29, 32]. While micromorphological samples remain preferable for a range of applications, examination of loose sediment also provides valuable information [62, 70, 71]. The inclusion of floated material opens up additional possibilities for complementary studies that provide broad-brush insights into long-term trends, facilitating integration with archaeobotanical data [30]. Such an approach is particularly useful for important legacy collections where sites can no longer be excavated and where sediment samples may not be available, or available in abundance.

It has generally been assumed that spherulites would not preserve in samples recovered via flotation, but spherulite counts from paired sediment and flotation samples at Ubaid period Tell Zeidan, just east of Abu Hureyra, demonstrate that while flotation does cause attrition, spherulites are recovered within the <1mm fraction of floated material in roughly the same *relative* proportions as those of sediments [30]. Phytoliths and starch grains are also recovered within the dust of flotation samples, even when the mesh used to collect samples is on the order of 1mm. Here spherulites are examined from flotation samples spanning the entire Epipalaeolithic and Neolithic sequence at Abu Hureyra.

### Archaeology of Abu Hureyra

Salvage excavations at Abu Hureyra, headed by Andrew Moore, took place in 1972 and 1973, focusing on seven trenches (A–G, Fig 1), exposing 8 m of stratified deposits [1]. In 1974, following completion of the Tabqa Dam, the site was submerged under water as Lake Assad was formed. The intensive sampling strategy adopted has allowed for detailed insights into both the Epipalaeolithic and Neolithic occupations of the settlements:

#### Epipalaeolithic (AH1)

**Fig 3:**
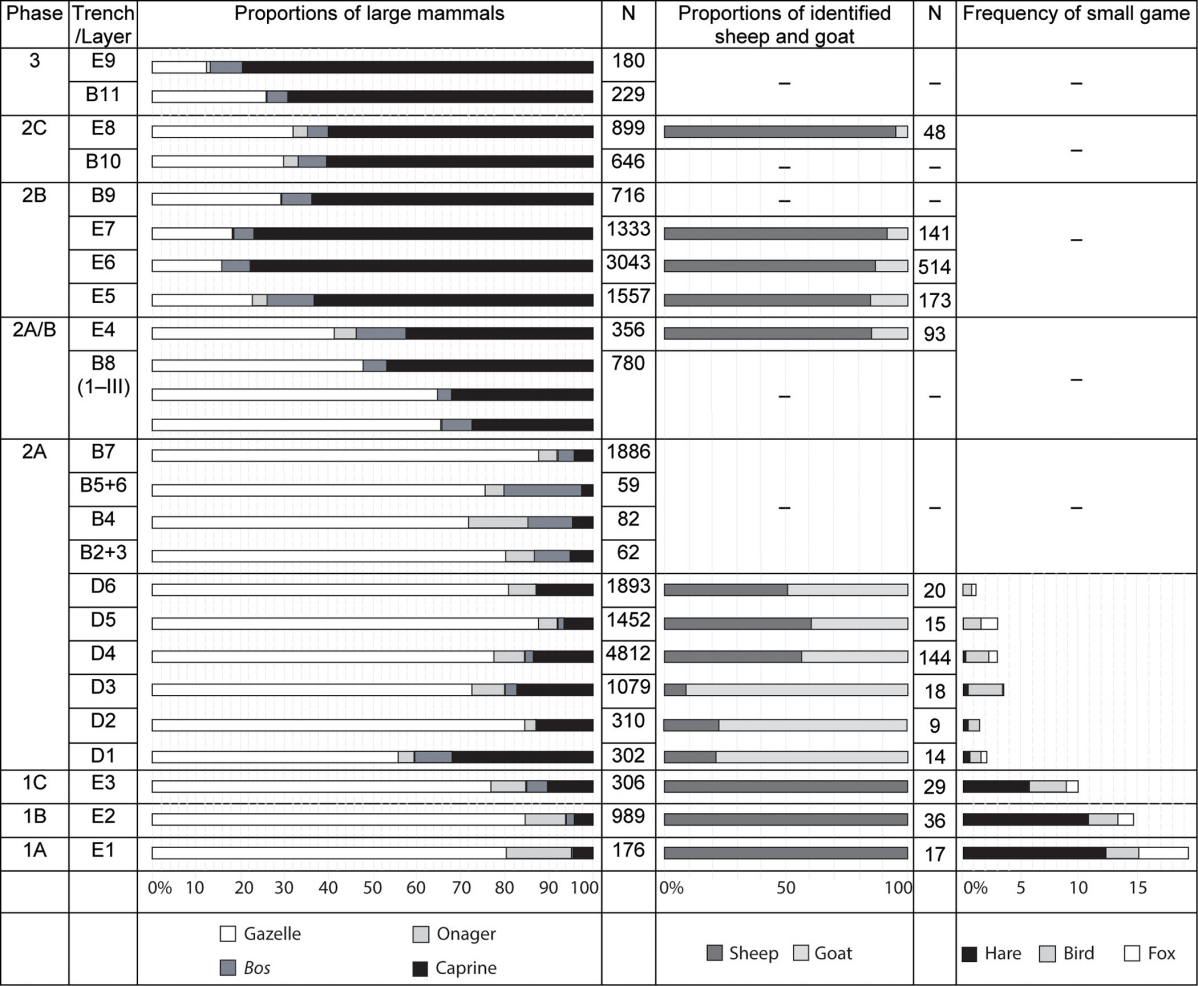
Bar charts illustrating relative proportions of large mammals (left), sheep and goat (center), and small game (right) within the Abu Hureyra sequence. All of the zooarchaeological data presented here were obtained from published reports produced by Legge and Rowley-Conwy [1, 76]. Following the method used by Colledge and Connolly [47] to explore published archaeobotanical data from Abu Hureyra, relative proportions and frequencies of fauna were obtained by measuring the length of the bars within Figs 13.6, 13.7, 13.8, and 13.1 from *Village on the Euphrates* [1]. Lengths of bars were measured in mm using low-power microscopy (10×) using the mid-point of lines at either end of the bar.– = no data available.

Excavations of the AH1 Epipalaeolithic occupation were restricted to Trench E (Fig 1C). Within the earliest occupation (phase 1A, ca. 13,300–12,800 calBP), several sub-circular, semi-subterranean pits (roughly 2–2.5 m in diameter) were joined to function as an occupational unit, allowing for internal movement (Table 1). Some of these pits contained querns. Post-holes indicate that the super-structure was roofed and opened out onto an external burned “bench” area that served as a work space (Fig 1D). During phase 1B (ca. 12,800–12,300 calBP), following abrupt climate change associated with the onset of the Younger Dryas [73, 74], rectilinear huts with thin clay or earthen floors were built atop the former pit-dwellings on level ground. Poplar (*Populus euphratica)*, willow (*Salix* sp.), tamarisk (*Tamarix* sp.), and ash (*Fraxinus* sp.) were commonly identified from occupational debris and interpreted by Roitel and Willcox as remnants of structures and fuel [1]. No deliberate burials were recorded within AH1, but Molleson observed isolated human bones exhibiting pathologies indicative of intensive and repeated use of a saddle quern for grinding [1]. Goitered gazelle (*Gazella subgutturosa)* bones dominated the Epipalaeolithic assemblage, but wild sheep/mouflon (*Ovis)*, onager (*Equus hemionus*), aurochsen (*Bos primigenius)*, hare (*Lepus capensis)*, fox (*Vulpes vulpes)*, birds (*Aves* spp.), and freshwater mussels were also present in all Epipalaeolithic levels (Fig 3) [1, 75]. Based on relative proportions of cranial and post-cranial bones, and mixed newborn–adult remains, Legge and Rowley-Conwy argued that gazelles were hunted *en masse* through non-selective seasonal culls of migrating herds, possibly using drives to trap and kill large numbers of animals [1, 3, 6]. Between phases 1A and 1C, hunting of small game consistently declined, concomitant with small but consistent increases in ungulate and *Bos* remains, a combined zooarchaeological signature that has more recently been linked with the beginning of early ungulate management elsewhere (Fig 3) [14, 27].

More than 120 plant taxa were identified from AH1 including wild cereals (rye/*Secale cereale* spp. *vavilovii*, *Triticum boeoticum*, *T*. *urartu)*, large seeded legumes (*Lens* sp., *Vicia/Lathyrus)*, tree fruits (including *Pistacia atlantica*, *P*. *khinjuk*, *Pyrus* spp., *Prunus* sp., *Celtis tournefortii)*, a variety of crucifers, small grasses and starch-rich foods such as *Bolboschoenus [Scirpus] maritimus/tuberosus*, cattail, bulrush, and water lily [1, 3, 4], collectively suggesting year-round occupation. During phase 1B, proportions of tree fruits dropped and small seeds from the woodland–steppe increased, a change Hillman linked with the cooler climate of the Younger Dryas [1]. The abundance of small grasses and legumes evident during Epipalaeolithic phases 1B and 1C (ca. 12,800–11,400 calBP) persisted into the PPNB (ca. 10,600–9,300 calBP). Following the debate with Miller regarding the dominant depositional pathway contributing to seed deposits at Abu Hureyra (and by extension elsewhere across Southwest Asia) [58, 59, 77], Hillman maintained that the earlier Epipalaeolithic assemblages reflected intentional plant gathering for human consumption while later assemblages included arable weeds associated with land disturbance, crop production, and crop processing [1].

#### Neolithic (AH2)

A sizeable Neolithic occupation was exposed in Trenches A–G. The Neolithic inhabitants of AH2 (phase 2A, ca. 10,600–9,300 calBP) expanded the settlement to roughly 8 ha and constructed more durable, multi-roomed, rectilinear mudbrick structures with plaster floors, marking a drastic architectural shift (Fig 1). Some of their construction removed the upper levels of the earlier AH1 occupation, leaving sparse remnants of an Intermediate occupation (ca. 11,400–10,600 calBP, Table 1) [1]. Villagers continued to hunt and gather wild taxa during phase 2A, but relied more heavily upon cultivated grains (rye, einkorn, emmer, bread wheat, 2- and 6-row barley, lentils, peas, vetch, field beans, and chickpeas) [5]. Gazelle hunting persisted as hunting of non-migratory wild fauna (wild cattle, pig, fox, hare) declined further and caprine remains increased. During the preceding Epipalaeolithic, wild sheep were the only caprids present at Abu Hureyra, but during phase 2A, goats suddenly appeared for the first time, as evidenced from remains in Trench D to the west of the mound (Fig 3). The landscape surrounding the site is not ideal terrain for wild goats, so their abrupt appearance (initially in greater relative proportions than sheep) is highly indicative of management, analogous to the appearance of goat in Cyprus [1, 12].

During phase 2B (ca. 9,300–8,000 calBP), villagers expanded the site further (16 ha), paralleling population growth at sites like Çatalhöyük and, for the first time, sheep and goat herding exceeded gazelle hunting, with sheep again becoming the dominant caprid [1] (Fig 3). Modest amounts of pottery appear for the first time towards the end of phase 2B. Site size and density dropped during phase 2C (7 ha, ca. 8,000–7,800 calBP) as space between buildings increased. Sheep and goat herding remained important as cattle and pig frequencies increased, and proportions of domesticated crops expanded relative to wild/weedy plants [1]. The site was abandoned around 7,800 calBP. Phase 3 includes evidence of late prehistoric and historic (including Byzantine) activity, and modern/historical burials on the surface that disturbed some of the earlier remains.

## Materials and methods

### Sample collection in the field

Archaeobotanical remains were initially recovered during the 1972 and 1973 excavations using froth flotation as part of a large-scale recovery effort [1]. The volume of sediment floated for each sample was measured in buckets (1 bucket ≈ 10L); sediment was then placed in a cylinder of water, mixed with a frothing agent (liquid detergent) and approximately 100 ml of kerosene (paraffin) to facilitate separation, and agitated with a stream of air bubbles to aid flotation. Archaeobotanical light fractions were collected with a 1-mm mesh prior to analysis at University College London (UCL) where the macro-botanical remains were studied under Hillman’s direction and remain well-documented and permanently curated [1].

### Gathering samples for spherulite analysis from the curated collection

In order to explore the presence of animals on-site and consider the varied uses of dung through time, flotation samples were collected from a variety of targeted archaeological context types, using descriptions assigned by excavators in the field as a guide, including: pyric features such as hearths and firepits (where remnants of fuel could reasonably be expected), as well as occupational debris from the pit dwellings, pit fill, plaster floors, an animal burrow, natural surfaces, and burials (the context type for each sample is listed within S1 Dataset). Permission to sample the curated light fractions (floated material) as part of this study was granted by UCL Institute of Archaeology Collections. Additional information regarding the original permissions to excavate at Abu Hureyra and export the flotation samples to the UK along with ethical, cultural, and scientific considerations specific to inclusivity in global research are included in (S1 File).

Light fraction samples were visually assessed at UCL to gauge the presence and approximate volume of the <1mm fraction (flotation dust) within each sample. When the amount taken constituted less than 10% of the amount available, between 0.25 and 1.0 ml of <1mm material was collected from each sample. This was done in order to preserve the integrity of the collection for future study. Observations on wood charcoal abundance and the presence of any modern contaminants, such as plant material or dung, were recorded.

### A new method for recording spherulites in isolated and cluster form

Spherulite analyses took place within the University of Connecticut Archaeobotany Laboratory. Samples were sieved using a 125-μm mesh to remove intact remains of charred plant remains that would artificially dilute the dust residue. All sieves were washed in vinegar, rinsed in water, and dried in an oven at 50°C for 10–15 minutes after each use to minimize cross-sample contamination. Slides and coverslips were cleaned using alcohol prior to use. One drop of mounting medium (85% Canada balsam, 15% methyl salicylate/wintergreen oil) was placed on a slide and weighed to 4 decimal places (other mounting media including Entellan could also be used). Roughly 0.0040 g of <125 μm dust was sprinkled over the mounting medium to obtain thin, even coverage. Excess powder was removed by tapping before the slide was reweighed to calculate sample mass. A coverslip was then placed over each sample and slides were held in a drying oven at 50°C for 24 hours prior to observation.

With the direct mount method used here, spherulites are not necessarily evenly distributed across the slide. When quantifying spherulites within loose materials, it is common practice to sub-sample a slide and, when remains are unevenly distributed, the use of random fields of view to count spherulites may not fully capture variation between samples. To offset this potential source of error, Gur-Arieh et al. [71, 78, 79] homogenize samples by vortexing and sonicating sediment within 2.4 g/ml sodium polytungstate (SPT, Sometu Ltd.) before mounting the suspension directly on a slide for observation. This has the benefit of more evenly distributing spherulites and facilitating accurate counts within random views. Given that: 1) this was an initial attempt to observe and quantify spherulite abundance throughout a sequence that included Epipalaeolithic levels, 2) numbers of spherulites in pre-Neolithic levels were presumed to be low before the study began, and 3) we hoped to examine whether dung clusters could prove helpful in assessing the potential for modern contamination, the direct mount method was chosen. Instead of sub-sampling, however, the entire area of the mounted material was observed and quantified using a continuous line moving up and down and left to right for full coverage. This was more time consuming, but helped minimize sampling error. Slides were examined using a Leica DM2700 materials microscope at 400× under cross-polarized and plane-polarized light.

Prior attempts at quantifying spherulites within loose sediments have recorded count values only. Here, all isolated spherulites and spherulites within very small 2-dimensional clusters were counted individually (Fig 2C and 2D). Recording accurate counts within larger 3-dimensional clusters is challenging, even when attempts to focus through the mass are made (Fig 2E); when encountered, the perimeter of each 3-dimensional cluster was drawn using NIS Elements software, and the area of each cluster recorded in mm^2^ in order to obtain a more objective, replicable measure. Spherulite counts were summed for each sample, as were the number and areas of clusters. Total counts and areas were then standardized to adjusted values per gram using the following equations:

$$\frac{\begin{array}{c} Total\;count\;of\;isolated\;\\ spherulites\;on\;slide \end{array}}{\mathrm{Mass}\;\mathrm{of}\;\mathrm{sample}\;(g)}=\mathrm{Adjusted}\;\mathrm{count}\;\mathrm{of}\;\mathrm{isolated}\;\mathrm{spherulites}/g$$

$$\frac{\begin{array}{c} Total\;area\;of\;spherulite\;\\ clusters\;\mathrm{on}\;\mathrm{slide}\;({\mathrm{mm}}^{2}) \end{array}}{\mathrm{Mass}\;\mathrm{of}\;\mathrm{sample}\;(g)}=\mathrm{Adjusted}\;\mathrm{area}\;\mathrm{of}\;\mathrm{clusters}\;({\mathrm{mm}}^{2})/g$$

In many instances, the original flotation samples had been recovered from enormous volumes of sediment, varying between 1 and 74 buckets per sample (each bucket ≈ 10 L; today sample volumes typically vary between 1 and 20L). Earlier studies indicate that while flotation causes attrition of dung spherulites, the relative proportions of spherulites observed between samples from both sediments and floated material remain similar [30]. It is not fully understood yet, however, how the initial sediment volume impacts spherulite counts within floated material. Because of this, two adjusted count and area values (mm^2^) were calculated and presented to enable transparent and fair comparison. The adjusted measures include per: “g of flotation dust” and “g of flotation dust/bucket,” whereby the former was divided by the number of buckets of sediment floated to calculate the latter value.

### Assessing the potential for sample contamination with modern dung

As any archaeologist in Southwest Asia is aware, domesticated animals frequently pass through archaeological sites, defecating as they go. Minute amounts of the inner matrix of dung pellets can yield thousands of spherulites invisible to the naked eye. Careful consideration of the potential for modern contamination is necessary, therefore, but remains largely unexplored within archaeological studies of spherulites. When flotation samples were examined within University College London, six samples contained modern plant material and/or modern sheep/goat dung pellets that could be seen with the naked eye. These samples were selected to serve as control samples to develop a signature for exploring potential contamination in other samples where dung was not clearly visible. These six samples were prepared and examined in the same manner as all of the other samples.

**Fig 4:**
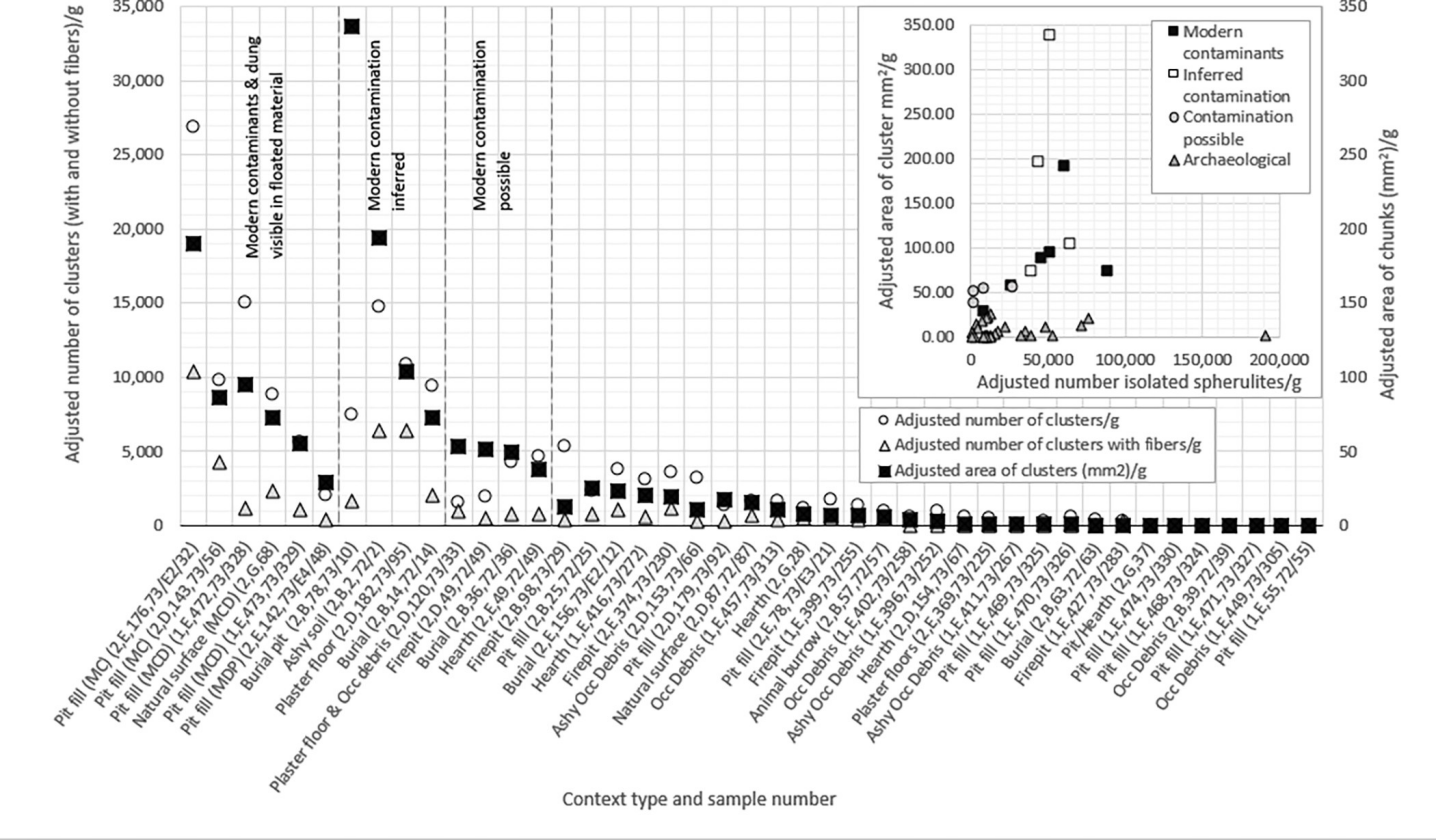
Scatter plot illustrating adjusted number and area of dung spherulite clusters within samples, highlighting samples with visible modern contaminants (MC), visible modern contaminants and dung (MCD), and samples with inferred or possible modern contamination. Inset: Scatterplot highlighting relationship between the isolated number of spherulites/g and the area of clusters mm^2^/g for contaminated, inferred contaminated and uncontaminated samples. No correlation between x and y variables: y = 0.0005*x* + 23.554, R^2^ = 0.0633.

**Fig 5:**
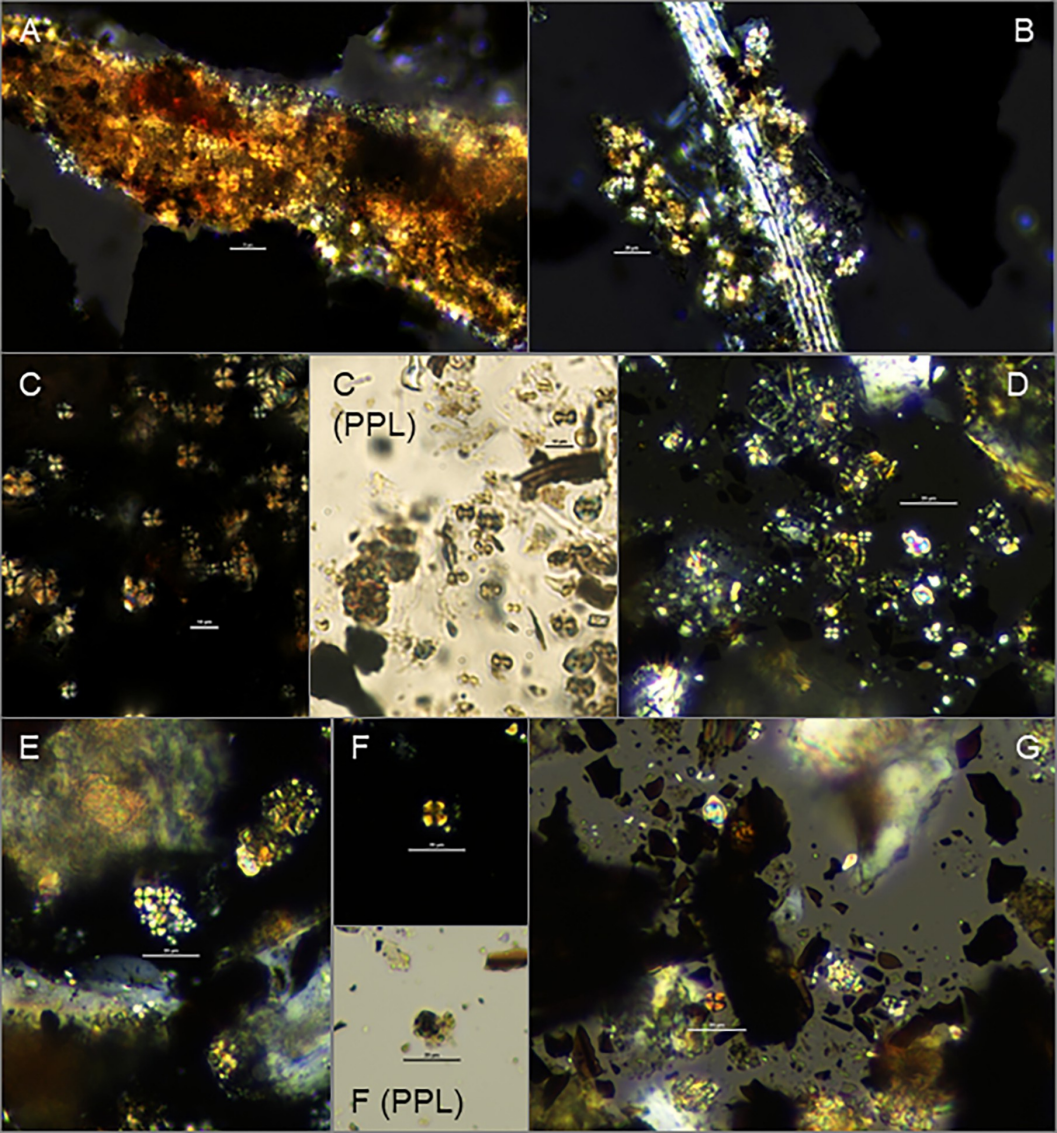
Photographs of spherulites documenting: A) and B) examples of spherulite clusters surrounding plant-based fibers within surface samples from Abu Hureyra known to be contaminated with modern dung (D,72,87 and D,73,95 respectively); C) modern sheep (*Ovis aries)* dung charred at 600°C for 2 hours revealing several large darkened spherulites indicating burning; D) general view of remains recovered from phase 2A/B plaster floor (E,369,73/225) rich in spherulites; E), F), G) a cluster of spherulites, an example of a darkened spherulite, and examples of isolated spherulites surrounded by abundant wood charcoal fragments respectively, all from Epipalaeolithic 1B firepit (E,427,73/283). Scale bar = 20μm in all images except for C, where bar = 10 μm. Images taken using cross-polarized light; PPL in images C and F indicates same view in plane polarized light.

Five of the six contaminated samples contained highly elevated adjusted numbers and areas of spherulite clusters (adjusted number of clusters: 5,614–26,957/g flotation dust, mean = 13,255/g, standard deviation = 8,390/g; area of clusters: 55–191 mm^2^/g, mean = 100 mm^2^/g, standard deviation = 53 mm^2^/g) (Fig 4). The adjusted counts of isolated spherulites were also high (9,167–88,929/g flotation dust, mean = 47,463, standard deviation = 27,674). Clusters were variably fragmented and many contained “fiber” inclusions that resemble uncharred, undigested plant material mirroring inclusions clearly present in modern, unburned comparative dung samples (Figs 2A, 2B, 5A and 5B). A sixth flotation sample (AH2, E 142, 73/E4/48, Phase 3) containing a single intact sheep/goat dung pellet, did not yield elevated clusters or counts of spherulites, suggesting that pellets only cause contamination when the outer surface is broken and the inner matrix is exposed. Data from this sample were viewed as “uncontaminated” and were included in the analysis of archaeological trends. The possibility of a relationship between adjusted isolated spherulite counts/g and the adjusted total area of clusters/mm^2^/g was explored using linear and logarithmic regression, and no correlation was found (linear: R^2^ = 0.0633; logarithmic: R^2^ = 0.1265) suggesting that a complex interplay of factors affect dung disaggregation, including the initial amount and timing of dung deposition (deposits are likely to experience over time), the extent of charring (which may enhance disaggregation), and the level of compaction (which may inhibit disaggregation).

Spherulite data from the five contaminated samples were used to develop a “contamination signature” to explore potential contamination in other samples. Samples with an adjusted area of dung >55 mm^2^/g *and* an adjusted count of clusters >5500/g were inferred to be contaminated. This threshold is a relative, rather than absolute, measure and is likely to vary between sites. The threshold resulted in the elimination of four samples (Fig 4). Three of these four samples had been excavated from the upper levels of Trench B and the fourth sample came from the upper levels of Trench D, likely representing surface contamination.

An additional four samples fell into an ambiguous area of “possible” contamination, meeting one, but not both, of the exclusion criteria. Three of these four “possibly contaminated” samples also came from surface sediments in the upper historical layers of Area E (AH2, B, 36, 72/36) and the uppermost layers of the side of the tell where Trench D was dug (AH2, D, 49, 72/49; AH2, D, 120, 73/33), further reflecting an anticipated level of elevated surface noise. These observations support the commonly held view that surface samples should be treated with caution. The fourth “possibly contaminated” sample came from a hearth (AH2, E, 49, 72/49) where an elevated dung signature aligns well with other pyric features. The remaining 29 samples deemed to be uncontaminated were excavated from well-defined features much lower down in the profile, and were presumed to reflect archaeological patterning. Data from these 29 sample were explored both with and without the four “possibly” contaminated samples. Since the inclusion of the four “possibly” contaminated samples did not affect the overall trends, but highlighted the potential for surface noise, they were kept in the dataset, resulting in 34 retained samples and nine rejected samples (S1 Dataset, Fig 4).

Post-depositional movements of spherulites resulting from human activity, insects, burrowing animals, or percolation also require careful consideration. The stratigraphy at Abu Hureyra demonstrated that newer occupants of Abu Hureyra routinely disturbed older sediments resulting in some mixing, a common phenomenon on mounded sites. Gerbils had created tunnels at Abu Hureyra and they too could have moved sediments containing spherulites, or even added to the spherulite record themselves. To minimize the impact of post-depositional factors, the samples examined here were carefully selected from well-delineated archaeological contexts representing floors, hearths, firepits, burials, and the inner fill and out-door work areas associated with the pit-dwelling [1]. An additional sample from a gerbil channel was also included in the analysis.

Given the high potential for modern contamination at the vast majority of archaeological sites across Southwest Asia, employing sample exclusion criteria will be important moving forward in order to securely isolate archaeological patterns. When attempting to assess modern contamination within loose materials, this is best done using direct mounts of unpulverized sediment or flotation material to examine the presence and abundance of dung clusters containing intact plant fibers. The fibers, which presumably represent organic material that is broken down through time or via charring, should also be visible within thin sections when present. Once potentially contaminated samples are eliminated, the wide range of quantification techniques that currently exist can be applied to facilitate counting. Studies examining spherulite movements within profiles would also be helpful.

## Deciphering dung within the archaeological remains

### Results

**Fig 6:**
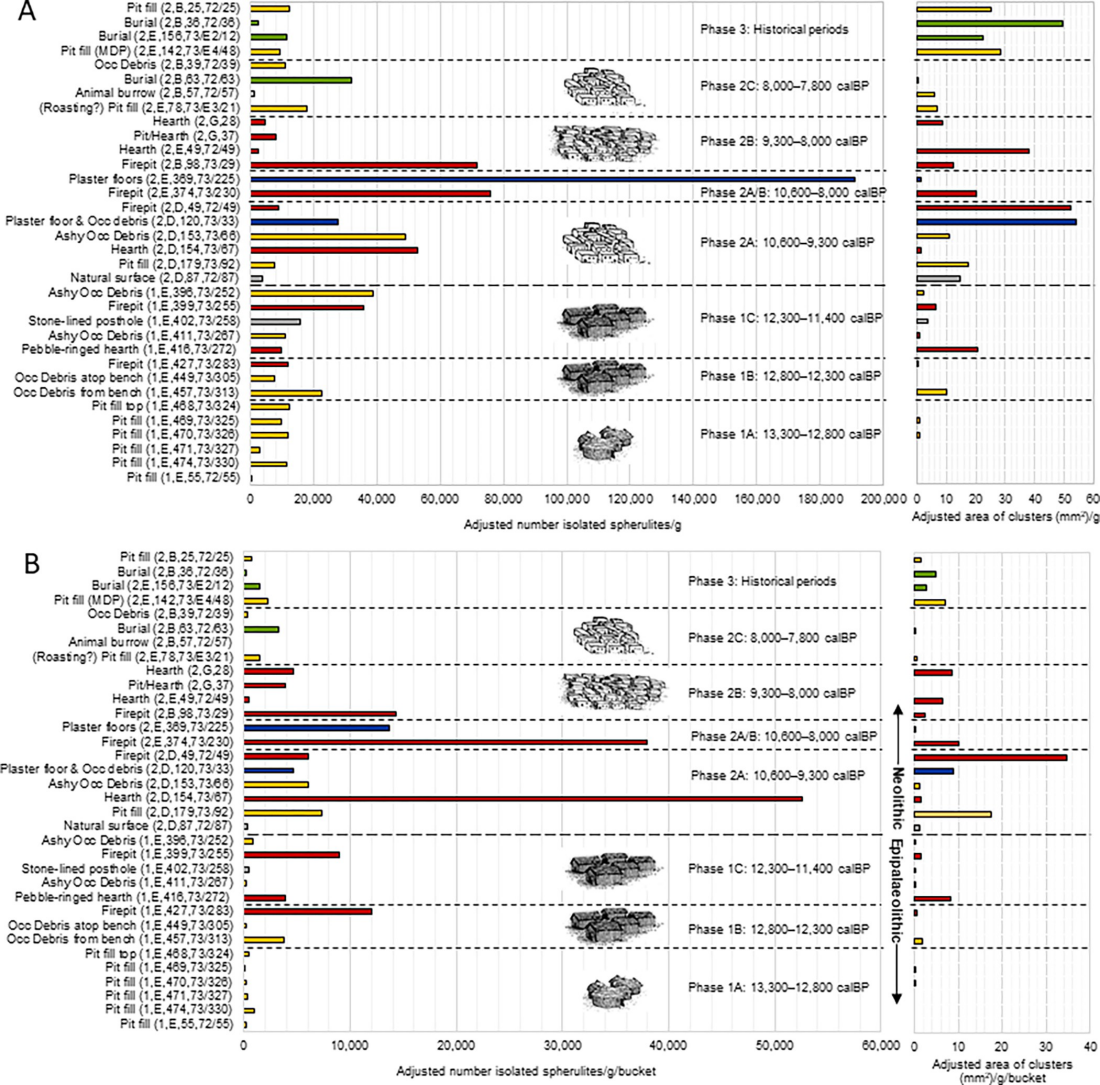
Bar charts illustrating the adjusted number of isolated spherulites (left) and adjusted area of clusters (right) per sample. The values presented are adjusted: A) per g of sample examined; and B) per g of sample examined/bucket of sediment to enable transparent comparison. Samples are organized from youngest (top) to oldest (bottom) following the Harris matrices from Abu Hureyra. Architectural and settlement trends for each phase of occupation are also illustrated. Key to color coding of bars: yellow = occupational debris and pitfill; red = pyric features (including hearths and firepits); blue = plaster floors; green = burials; grey = other (context details, including trench, level, and feature type, are noted in the y-axis sample labels).

The full dataset detailing the adjusted counts of isolated spherulites and the adjusted numbers and areas of spherulite clusters observed in each of the 43 <1mm flotation samples examined is provided within the S1 Dataset alongside data detailing the archaeological context, the mass of the sample examined, the volume of sediment originally floated for each sample, and observations of wood charcoal fragments. Data from samples representing archaeological trends are illustrated in Fig 6.

Dung spherulites are present in varying relative quantities throughout the entire stratigraphic sequence spanning Epipaleolithic and Neolithic occupations (Fig 6). The long-term trends within both adjusted measures (Fig 6A: per g of flotation dust, and Fig 6B: per g of flotation dust/bucket of sediment) were broadly similar, albeit with some notable exceptions highlighted below. Such an early occurrence of animal dung within the Epipalaeolithic, when the site was occupied by hunter-gatherers, requires careful consideration (Fig 6). Various depositional pathways and explanations of the relative differences in spherulite abundance through time and between samples and feature types are presented below, alongside a discussion of associated archaeological data.

### Discussion

Much of the occupational debris from Abu Hureyra represents time-averaged accumulations of remains within each occupational phase, resulting from a gallimaufry of repeated and diverse activities [1, 48, 80]. Several of the archaeological contexts examined here represent features that were well-defined in the field, recording shorter-term examples of past activity, including floors and pyric features, such as firepits and hearths (although both can be subject to complex cycles of repeated use and cleaning) [81].

The sample yielding the greatest concentration of isolated spherulites came from blocky remnants of a Neolithic plaster floor that had been renewed several times within a 3-room house (AH2, E369,73/225, phase 2A/B) (Figs 5D and 6). The use of dung to prepare floors is well-documented, both ethnographically and during the Neolithic, when clear evidence for animal tending exists [79, 82] and excess dung was presumably put to practical use. Dung is typically mixed with water and spread out, serving as either the main floor material or as a substrate to a plaster floor [82]. Archaeological parallels include Late Neolithic Makri in Greece, where a 3–5 mm layer of burned dung lay between two lime plaster floors [83] and late PPNB Tell Seker al-Aheimar, Syria [31]. Given that the floor layers were collected via bulk sampling, and also include some occupational debris, values adjusted for original sediment volume artificially dilute the signature (Fig 6).

Conversely, occupational debris from the earliest Epipalaeolithic 1A pit-dwellings (13,300–12,800 calBP), beginning approximately 5 m below the modern-day surface, yielded low isolated spherulite counts and virtually no clusters. The relative paucity within these time-averaged accumulations was amplified even further when the original sediment volume floated was considered, rendering their presence barely perceptible in the bar chart (Fig 6B).

From the >1mm fraction of floated remains, Hillman described abundant, black–brown fragments (diameter ≈ 2mm) within many Epipalaeolithic samples. While acknowledging their rather enigmatic nature, Hillman suggested that they may represent infant feces based on their shape, texture, and ethnoarchaeological associations with! Kung and Gidjingale forager waste-disposal practices [4, 84, 85]. Their presence is firmly associated with the original settlers of the site, but since their identify has not been confirmed using fecal biomarkers such as coprostanol, it is not fully clear what they represent. It is possible that intensifying efforts to develop methods for identifying food lumps will prove helpful in further exploring their identity [86, 87]. Little is known about spherulite production in human infants, but given their high calcium demands and their isotopic trophic association with carnivores, it is reasonable to assume that spherulites would be sparse to absent in infant feces [88]; a similar pattern is documented in sheep, whereby lambs that have not yet weaned do not produce spherulites; older sheep do owing to their different diets and calcium needs [65].

By Epipalaeolithic phase 1B (ca. 12,800–12,300 calBP), spherulite presence is more robust in both isolated and cluster form and in both adjusted measures, but particularly so when adjusted for original sediment volume (Fig 6B). Dung is present within a well-delineated firepit (AH1, E, 427, 73/283) and within a sample from the outdoor bench work area immediately outside of the dwelling (AH1, E, 457, 73/313), where the earliest spike of dung clusters was recorded (Figs 5 and 6). This abrupt appearance contrasts with the relative paucity of spherulites within earlier samples but also with the phase 1B sample that had accumulated directly atop the bench (AH1, E,449,73/305), suggesting that spherulites had not moved down within the profile.

The presence of darkened spherulites within multiple pyric features throughout the sequence confirms that dung was burned between 500 and 700°C beginning as early as the Epipalaeolithic 1B phase (12,800–12,300 calBP, Fig 5F), a practice that continued through Epipaleolithic 1C (12,300–11,400 calBP) and persisted into the Neolithic, marking the earliest use of dung fuel. The elevated concentration of spherulites within pyric features was particularly noticeable when spherulite values were adjusted for initial sediment volume, owing to the small size of many of the pyric features. Given the abundance of microscopic wood fragments in many of the slides examined here (Fig 5G), combined with general observations of abundant wood charcoal in many of the flotation samples (S1 Dataset), wood remained the dominant fuel throughout the Epipalaeolithic and Neolithic, with dung being used as a casual supplemental fuel source [1, 59]. Not all hearths contained high spherulite values, however, particularly during phase 2B, when fuel use practices may have shifted (although spherulites continued to be present). Possible reasons for these changes are considered below.

The sample from the fill of an intrusive gerbil burrow that penetrated the phase 2C remains yielded relatively low spherulite counts (Fig 6). While this single measure cannot be used to discount the impact of bioturbation across the entire site, it is important to note the samples examined here were excavated from archaeologically-distinct features that were determined to be intact in the field. While taphonomic processes likely introduce “noise” into any spherulite dataset, the repeated and notable relative differences in spherulite abundances between pyric features, floors, and other context types that were observed here conform with additional archaeological data sources in a meaningful way, throughout the entire 8m deep sequence, suggesting that the spherulites reflect real behavioral trends.

### Considering the presence of animals on-site

The presence of dung at Abu Hureyra, particularly during the Epipalaeolithic, begs the question of how the dung got there. Sparse concentrations of spherulites are present in phase 1A (ca. 13,300–12,800 calBP) but, until further spherulite studies are conducted at contemporary sites, they remain difficult to interpret. By phase 1B, when inhabitants make the dramatic shift to more durable rectilinear architecture, burned dung is present both within a firepit (largely in the form of isolated spherulites) and isolated spherulites and dung clusters are particularly noticeable within the sample collected from the work area outside of the rectilinear structure (Fig 6). Through collective consideration of the architectural, zooarchaeological, fuel, and spherulite data, we hypothesize that live animals were held outside the hut as early as 12,800–12,300 calBP, with hunter-gatherers disposing of accumulating piles of excrement as a convenient fuel that supplemented wood burning. Such co-presence of people and animals on-site likely involved small numbers of animals and could have been short-term, providing a means for delaying slaughter during temporal gaps in gazelle availability and further enabling year-round occupation of the site. Any arrangement beyond several days would have necessitated low-intensity care or tending through the provision of food and water.

It is currently unclear which animal deposited the dung. Some scholars have used macro-botanical or phytolith data to explore animal diet, potentially providing an additional avenue to explore species, but this approach rests firmly on the assumption that plant remains recovered were deposited solely via dung-depositional pathways [61]. A detailed consideration of plant data from Abu Hureyra lies beyond the scope of this paper, but it is clear that the archaeobotanical assemblages represent variable mixtures of human-consumption and wood- and dung-based fuel pathways, and that the relative contribution of each pathway varied between samples and time periods, rendering it difficult to cleanly isolate animal diet from plant data without additional study [61, 89]. Furthermore, even if purely dung-derived deposits are secured, modern studies of goitered gazelle and mouflon diet, together with historical knowledge of extinct aurochsen, indicate substantial overlap in the range of grasses grazed, and opportunistic browsing of forbs, leaves, bark, and twigs as conditions permit or seasons require [90–93].

The zooarchaeological data provide more reliable clues. The Epipalaeolithic faunal record from Abu Hureyra documents large proportions of goitered gazelle, alongside smaller proportions of onager, aurochs, wild sheep, hare, fox, and birds (Fig 3). Spherulites cannot be identified to taxa, but since gazelles produce spherulites (Fig 2), it is theoretically possible that their dung was burned, as originally suggested by Miller [58]. Gazelles often mark territory with their dung, defecating repeatedly in the same area, creating dung piles that are easy to collect. Wood remained the fuel of choice throughout the sequence, particularly during the Epipalaeolithic, and wood sources are thought to have been widely available around Abu Hureyra for the entire occupation [1], so there appears to be little incentive for the occupants of Abu Hureyra to transport these piles to the site for burning, especially if the piles were distant from the settlement. It is also unlikely that gazelles were captured for on-side holding. They have a loose social structure and the males, in particular, can become very aggressive when held captive, making them challenging animals to tend [94].

Patterns within the Epipalaeolithic faunal sequence from Abu Hureyra point to an alternative, more likely source: wild sheep. Such an assertion would have been less probable several decades ago when Miller and the Abu Hureyra team were writing, but recent studies elsewhere provide growing support for early attempts at small-scale caprid tending prior to the advent of morphological changes in bone. Steady decreases in reliance upon small game between phases 1A–1C at Abu Hureyra cooccur with small increases in *Ovis* sp. and *Bos* sp. relative to large mammals (Fig 3), a subtle, yet notable zooarchaeological signature associated with incipient management of caprids in the southern Levant [14], Zagros [38] and Central Anatolia [27]. Using measurements of bone size and temporal shifts in the Abu Hureyra faunal sequence, Legge and Rowley-Conwy argued that “caprine domestication was accomplished” by Neolithic phase 2A (ca. 10,600–9,300 calBP) [1]. Goats suddenly appear during phase 2A (possibly introduced), but sheep were present in low numbers as early as phase 1A, and increase steadily thereafter until they dominate the assemblage in phase 2B (Fig 3). Given that experiments in tending of varying intensities likely predate the appearance of morphological markers of domestication during phase 2A, and the zooarchaeological data from Abu Hureyra suggest emerging caprid management, morphologically wild sheep are strong contenders for earlier on-site presence. The behaviors of wild sheep, with fixed-membership herds based on a male hierarchy, makes them much more amenable to tending than gazelle, providing a necessary precursor to domestication. Aurochsen are also present during the Epipalaeolithic and, theoretically, could have contributed dung, although behavioral models suggest that they would be much more difficult to capture live than wild sheep in the earliest experiments with tending [94].

No structural evidence for penning was documented outside of the phase 1B structure where the dung was found at Abu Hureyra, paralleling finds from Neolithic Pinarbaşı, Çatalhöyük, Boncuklu, and Aşıklı Höyük, where phytoliths and micromorphology mark the earliest examples of on-site animal keeping in open areas recorded to date [27–29, 39]. Using multiple lines of evidence from the Level 5 occupation at Aşıklı Höyük, Stiner et al. argue that wild lambs and kids were captured and raised for several months prior to slaughter, as a form of small-scale live “catch-and-grow” meat storage that provided quick returns for low investment, between 10,450 and 9450 calBP [42]. Over a span of 1000 years, the relationship between people and caprids intensified at Aşıklı Höyük, culminating in large-scale herding, much as it did at Abu Hureyra. The amount of time that animals were kept alive on-site by hunter-gatherers at Abu Hureyra is unclear but was likely short term. As stated earlier, any arrangement beyond a couple of days would require some form of tending.

Published zooarchaeological data from across the region indicate that shifts to domestication took place earlier in the Euphrates than in Central Anatolia, with domestic sheep and goat in Southeastern Anatolia by 10,500 calBP and reduced sexual dimorphism in cattle around the 11^th^ millennium calBP [20]. Zeder asserts that the initial management of sheep and goat likely began up to 11,000 years ago in the highland regions of the eastern Taurus and northwestern Zagros Mountains and that the initial transition from hunting to herding may “reach back to about 12,000 to 13,000 calendar years ago” [35]. Evidence from Abu Hureyra, suggesting that animals were kept on-site between 12,800–12,300 calBP, pre-dates the Aşıklı Höyük find, as expected given the differing trajectories between the two regions and, while roughly 2000 years earlier, conforms with existing expectations for the broader region. Existing work has tended to link animal management solely with Neolithic people, making this a novel observation, but it is important to stress that few empirical attempts have been made to track tending during the Epipalaeolithic, based primarily on a paucity of reliable methods, but also a weakening assumption that hunting was the *only* strategy used. Echoing Higgs and Jarman’s 50-year old call, it is fair to question this assumption and further work on Epipalaeolithic sites is needed [34]. The observations made here raise the question of whether shifts in animal management pre-date pre-domestication cultivation or whether they emerged contemporaneously.

Outside of the middle Euphrates, many other examples of early small-scale tending are beginning to emerge. At early Neolithic Bestansur in the Shahrizor Plain of Iraq of the western Zagros foothills, for example, spherulites and omnivore coprolites document close proximity of animals and people within the settlement close to 10,000 years ago [33]. Beyond Southwest Asia, additional studies highlight the common practice of holding small numbers of animals immediately outside of a dwelling without penning, as people embarked on a transition from predominantly hunting to herding. In her examinations of horse domestication at Botai, Kazakhstan, Olsen recorded elevated levels of nitrogen and stanols specific to horse adjacent to homes, indicating that livestock were frequently kept there [26]. In northwest Siberia at I͡Arte VI, Anderson et al. similarly observed elevated levels of 5β-stanols and phosphate levels within palaeosols immediately outside the pit houses, indicating the presence of *Rangifer* (reindeer). They argue for a small number of animals (on the order of 5–6 head) who may have been “relied upon to willingly stay close to the camp without the use of confining structures” or were tethered since no evidence for pens, corrals, or physical structures exist [24]. Combined consideration of zooarchaeological data and dung from I͡Arte VI indicate a complex interplay of hunting and herding of reindeer as people transitioned away from hunting only [24]. Given these widespread examples, it is likely that formal penning was not used until more intensive, longer-term forms of tending began.

When attempting to track early evidence for a shift in the relationship between people and animals, behavioral change can be subtle and difficult to detect archaeologically, requiring multiple lines of evidence. This point was stressed by Gron and Rowley-Conwy [95] who used isotopic analyses to successfully observe the earliest beginnings of small-scale *Bos* herding and the expansion of agriculture within forested areas in southern Scandinavia, after agriculture had become well-established elsewhere and was beginning to spread into northern Europe. Olsen echoes this point through her work on horse, similarly arguing for a multi-faceted approach, noting that as secondary evidence reaches a critical mass, it becomes more and more likely that some form of oversight existed [26]. Co-presence is a necessary pre-requisite for the type of long-term tending that appears later on at Abu Hureyra. The relative changes in spherulite abundances observed throughout the sequence here, combined with knowledge of fuel use and faunal assemblages, all point to co-presence beginning in the Epipalaeolithic, when the site was occupied by hunter-gatherers. While it is not known whether the animals at Abu Hureyra were there willingly or not during Epipalaeolithic phase 1B, or how long they were there, multiple lines of evidence suggest that they were on site for sufficient periods of time to leave an elevated spherulite signature directly outside the Epipalaeolithic 1B dwelling—and that the dung was then used as a conveniently available, supplemental fuel. Considerations of dung (observed within thin sections and loose sediment) combined with zooarchaeological signals at other sites will help determine whether animals were commonly held on Epipalaeolithic sites across Southwest Asia. Additional studies of stanols will also undoubtedly prove useful. Using principal components to analyze proportions of various stanols within modern animal dung from 10 different taxa, Harrault et al. [96] were able observe distinct stanol signatures that enable archaeological identification of taxa beyond broad carnivore/omnivore/herbivore categories. Given that stanols preserve well over time, bind to organic matter, and have low water solubility, there is a possibility that they preserve in flotation materials alongside spherulites. Future work will explore whether samples from Abu Hureyra contain 5β-stanols, nitrogen, and phosphates, allowing for greater insight into the species present [85, 96].

### Intensifying interaction

Observations of dung spherulites throughout the entire sequence of occupation at Abu Hureyra place the Epipalaeolithic samples within a broader temporal context of change, helping reinforce the emerging patterns evident throughout the occupation. During the Neolithic phases 2A (10,600–9,300 calBP) and 2A/B (10,600–8,000 calBP) at Abu Hureyra, when animal herding was well established, there is clear evidence for use of dung as a construction material within plaster floors and continued use as a fuel, highlighting the ubiquity of dung as a raw material. The amounts of spherulites recorded in general occupational debris and pit fills also increases relative to the earlier samples. Presumably, this greater abundance and ubiquity reflects the greater availability of dung, linked to a greater reliance upon domesticated animals.

Spherulite abundances steadily increase through time but, by the latter part of phase 2B (9,300–8,000 calBP), the abundance of both isolated spherulites and clusters drops in pyric features relative to phase 2A by several orders of magnitude, a pattern that persists throughout Late Neolithic phase 2C (8,000–7,800 calBP) (Fig 6). Changes in fuel preferences or hearth cleaning practices could explain this drop, but a shift in animal management strategies could also play a role. If wood remained the preferred fuel, as the dominance of wood remains within pyric features suggests, *and* dung was used as a convenient supplement when available on-site, or in close proximity to the site (rather than being imported to the settlement from some distance), the general trends in spherulite abundances could provide a broad-brush reflection of long-term fluctuations in on/near-site animal densities. The drop in spherulites within pyric features beginning in phase 2B corresponds with a dramatic increase in human occupation and, for the first time, greater reliance upon domesticated animals relative to hunted taxa, a pattern that persists into phase 2C [1]. While seemingly paradoxical, as reliance on sheep and goat intensified, on-site management of large numbers of animals would become untenable, necessitating large-scale movements of sheep and goat herds away from the settlement, thereby reducing the immediate availability of dung on-site.

As the spherulite signature lessens, domesticated crops become more prominent within the archaeobotanical record, a trend that continues into Neolithic phase 2C [1]. Again, a detailed reflection on the plant remains from Abu Hureyra lies beyond the scope of this paper, but the results generated here highlight how the relative contributions of plants representing human- and animal-related consumption patterns vary between archaeological context types and shift through time. A more detailed study of spherulite abundances across a wider range of archaeological context types within the Neolithic levels at Abu Hureyra is planned for the near future. Based on studies of spherulites at other sites across northern Syria dating to later time periods, it would appear that fuel selection choices and depositional processes become even more complex during the Ubaid, Chalcolithic, and Bronze Ages onwards as fuel demands increase and morph helping to drive craft specialization, the availability of wood resources decreases, and the range of activities performed shifts dramatically to include metal working [30, 54]. Collectively, the range of activities performed in the past, combined with changes in construction materials and food and fuel-based economies, shape the differential contributions to archaeobotanical assemblages through time and space, underscoring the need to consider plant deposition on a sample-by-sample basis. We would argue that such considerations are essential, moving forward, given that the range of questions that can be reliably addressed using archaeobotanical data rests firmly upon a secure understanding of sample context and depositional pathways.

## Summary and conclusions

Dung spherulites were observed in both isolated and cluster form within the <1mm fraction of curated flotation samples from the Epipalaeolithic and Neolithic sequence at Abu Hureyra. Dung spherulites were present in every sample in varying frequencies. A signature was developed to exclude samples that were likely contaminated with modern dung; use of a similar approach may be helpful for future spherulite studies elsewhere given the high potential for contamination within archaeological sites across Southwest Asia, particularly in surface levels.

When considered alongside architectural, fuel, and zooarchaeological data, observations of ancient dung within samples from Abu Hureyra help deepen our understanding of the shifting relationship between people and animals. The data presented here mark the transition from early, short-term, on-site animal tending during the Epipalaeolithic 1B phase to full scale, off-site herding during Neolithic 2B. The elevated presence of dung immediately outside of an Epipalaeolithic 1B dwelling cooccurs with an abrupt shift to rectilinear architecture, increasing proportions of wild sheep and aurochs in the faunal record, reduced emphasis on small wild game, and early use of dung fuel as a supplement to wood within a firepit. Various possible explanations for these trends were considered. Collectively, spherulite, fuel, architectural, and zooarchaeological data suggest that live animals, possibly sheep, were held on-site at Abu Hureyra by hunter-gatherers during Epipalaeolithic phase1B, dating between ca. 12,800–12,300 calBP, serving as live meat storage when gazelle were not available. This practice was small-scale and remained firmly embedded within a culture of gazelle hunting that continued through phase Epipalaeolithic 1C. Tending small numbers of animals immediately outside of dwellings appears to have been a common strategy used by people as part of the early transition from hunting to herding across Southwest Asia and beyond. This marks the earliest occurrence of such tending, raising the questions of whether early animal tending occurred before or alongside early cultivation, rather than developing later.

By the Neolithic (phase 2A, 10,600–9,300 calBP), when herding was well established at Abu Hureyra, dung continued to be used as a fuel and was also used to prepare plaster floors, a practice that was widespread during the Neolithic across Southwest Asia. Spherulites appear more abundant in general occupation debris, reflecting the greater availability of dung. Wood appears to have remained the fuel of choice throughout the sequence, however, with dung being used as a supplement (with increasing intensity during phase 2A). By Neolithic phase 2B, spherulite abundances drop dramatically within pyric features, at a time when sheep and goat remains begin to dominate the faunal assemblage, exceeding gazelle remains for the first time. Many factors affect spherulite abundance within individual features, but if dung was burned as a convenient means of eliminating on-site accumulations during the Epipalaeolithic and Neolithic, then general shifts in spherulite abundance through time could serve as a proxy for the intensity of on-site animal presence and tending, whereby increasing numbers of livestock were supported outside the main habitation area beginning towards the end of phase 2B (9300–8000 calBP). This relationship would not necessarily hold during post-Neolithic periods when populations increase, fuel demands change in relation to evolving craft economies, and deforestation reduces wood availability necessitating importation of a variety of fuels [30, 53, 54].

Over the past decade, a growing body of integrated zooarchaeological and dung studies, has allowed the incipient stages of animal management to be observed, highlighting some of the diverse strategies that people used to interact with wild animals. This collective work is shifting the lens further and further back in time. Observations from Abu Hureyra indicate small-scale on-site animal keeping during the Epipalaeolithic between 12,800 and 12,300 calBP, by people continuing to live a hunter-gatherer lifestyle. Additional studies of dung distributions across Epipalaeolithic sites, particularly in the Euphrates valley and Taurus-Zagros arc, are needed to further explore the temporal and geographic range of animals on sites and to consider the full range of animal-related strategies used by people prior to the large-scale abandonment of hunting that occurred later on. This study highlights the utility of well-curated archaeological collections, particularly from important sites that cannot be re-excavated or from regions that are not currently accessible. Fifty years on, the samples from Abu Hureyra still have much to tell us.

## Supporting information

S1 Dataset List of samples discussed in the paper, including archaeological context summaries, sample age, volume of original sediment sample, mass of mounted flotation dust sample, and adjusted values per g of flotation dust and per g of flotation dust/bucket of sediment. (XLSX)

S1 File Inclusivity in global research statement with excavation permits. (PDF)
