# Supplementary material for: Epipalaeolithic animal tending to Neolithic herding at Abu Hureyra, Syria (12,800–7,800 calBP): Deciphering dung spherulites
Source: PLoS One. 2022 Sep 14;17(9):e0272947. doi: 10.1371/journal.pone.0272947 (PMC9473395; doi:10.1371/journal.pone.0272947)
Supplement: S1 File — (PDF) [file pone.0272947.s002.pdf]

# Inclusivity in global research

PLOS' policy on inclusivity in global research aims to improve transparency in the reporting of research performed outside of researchers' own country or community and ensures that PLOS publications reporting global research adhere to high standards for research ethics and authorship. Authors of relevant research articles may be asked to complete the questionnaire below, which outlines ethical, cultural, and scientific considerations specific to inclusivity in global research. This questionnaire may be requested when researchers have travelled to a different country to conduct research, if research uses samples collected in another country, research with Indigenous populations or their lands, or if research is on cultural artefacts. Researchers travelling to another country solely to use laboratory equipment will not normally be required to complete the questionnaire. However, the questionnaire can be requested at the journal's discretion for any submission – if you have been requested to complete this questionnaire by the PLOS journal you submitted to, please do so.

Please complete the questionnaire below and include this as a Supporting Information file with your manuscript. Note that if your paper is accepted for publication, this checklist will be published with your article in the supporting information files. Please ensure that you reference the checklist in the main body of your manuscript. We suggest adding a subsection 'Inclusivity in global research' to your Methods section and adding the following sentence: "Additional information regarding the ethical, cultural, and scientific considerations specific to inclusivity in global research is included in the Supporting Information (SX Checklist)"

The questions have been designed to be applicable to a wide range of study types, and there are subsections for both human subjects research and non-human subjects research. If any of the questions are not relevant to your research please mark them as "N/A" as appropriate.

## Ethical considerations, permits and authorship

*This section is applicable to all research types.*

Provide details as to who granted permissions and/or consent for the study to take place in the Methods section of your manuscript. This should include the names of **all** ethics boards, governmental organizations, community leaders or other bodies that provided approval for the study. If individuals provided approval refer to these people by their role or title but do not list their name(s).

| |
|---|
| Reported on page number: 18 |

If there were any deviations from the study protocol after approval was obtained please provide details of these changes in the Methods section of your manuscript.

| |
|---|
| Reported on page number: Not applicable |

Did this study involve local collaborators that are residents of the country where the research was conducted or members of the community studied? If you do not have any authors from said communities, please provide an explanation for this below.

| |
|---|
| No. The samples examined here were excavated from Abu Hureyra, Syria, between 1972 and 1973, and are currently curated at University College London, UK. The officials of the Syrian Directorate General of Antiquities and Museums (DGAM) who authorized the Abu Hureyra excavation project 50 years ago are now deceased. A representative from the DGAM was present on the site at all times to oversee the excavation and curation of the materials recovered. As far as we are aware, there are currently no Syrian scholars with the specific expertise required to collaborate substantively in this research. |

Everyone listed as an author should meet PLOS' criteria for authorship and all individuals who meet these criteria should be included in the author byline, rather than the acknowledgements. Authorship criteria is based on the International Committee of Medical Journal Editors (ICMJE) Uniform Requirements for Manuscripts Submitted to Biomedical Journals - for further information please see here: https://journals.plos.org/plosone/s/authorship.

## Human subjects research (e.g. health research, medical research, cross-cultural psychology)

Did you obtain written informed consent from a representative of the local community or region before the research took place? How did you establish who speaks for the community? Details of written informed consent obtained from study participants should be reported separately in the Methods section of your manuscript.

| |
|---|
| Not applicable: no human subjects. |

How did members of the local community provide input on the aims of the research investigation, its methodology, and its anticipated outcome(s)?

| |
|---|
| Not applicable. |

When engaging with the local community, how did you ensure that the informed consent documents and other materials could be understood by local stakeholders?

Will the findings of the research be made available in an understandable format to stakeholders in the community where the study was conducted (e.g. via a presentation, summary report, copies of publications, etc.)? Please provide details of how this will be achieved.

Not applicable.

## Non-human subjects research using specimens/ animals collected as part of the study, or those housed in archival collections. Examples include archaeology, paleontology, botany and zoology.

Did the permission you obtained from a local authority to perform the study include an agreement on access to outputs and benefit sharing? This may include procedures to enable fair distribution of the benefits and resources arising from the research performed. Please include any details of Prior Informed Consent and Benefit Sharing Agreements obtained. These may be required by field-specific regulations, for example the Convention on Biological Diversity (CBD) and the associated Nagoya Protocol.

The permission granted by UCL did not include such a provision. However, all partipants in research on material from Abu Hureyra are expected to publish their results. This paper represents just such an example. Our manuscript was submitted to *PlosOne* owing to our commitment to open access publication. All data included in this study are available through published sources or within the Supplemental dataset.

If the material used in your study was imported, please A) provide the year it was imported and B) indicate whether permits were obtained to import/export the materials used, C) provide details of any permits obtained. If this information is not available, please indicate this.

A) The flotation samples were imported to the United Kingdom from Syria in 1972 and 1973. For this study, the <1mm fraction of floation samples was sub-sampled and transported to the University of Connecticut, USA in June 2019.
B) Permission to export these samples from Syria was given by the DGAM at the time. In order to transport the flotation dust examined here from the UK to the USA, a USDA soil permit was used.
C) A copy of the original excavation permit is attached. An associated Decree of the Council of Ministers, Syrian Arab Republic, number 295, issued on 2.12.1969 (also attached) provides the underlying legal authority for the disposition of the material excavated at Abu Hureyra. Both the original permit and the Decree are in French. English translations are also provided. The USDA Permit to Receive Soil (P330-17-00081, valid until 16 April 2020) was issued to Alexia Smith.

If you used archival specimens, please state how the material used in your study was acquired by the institute it is held in and provide details of any permits obtained for the original excavations/ sample collection. If this information is not available, please indicate this.

The samples were donated to the Institute of Archaeology, University College London by Andrew Moore, the excavator of Abu Hureyra, with the permission of the DGAM. The remains are permanently curated there. Please see note above regarding permits for export and associated permissions. Alexia Smith requested permission to study the collection at UCL in May 2019. Permission was granted in June 2019.

How was the potential cultural significance of the materials collected in your study to local communities considered in your research design? Were Indigenous peoples and/or local researchers and institutions involved with archaeological excavations / collection of specimens? If so, please provide a description of their involvement.

As stated above, a representative of the Syrian Directorate General of Antiquities and Museums (DGAM) who authorized the Abu Hureyra excavation, was present on the site at all times to oversee the excavation and curation of the materials recovered. The inhabitants of the modern village of Abu Hureyra did the bulk of the excavation work and also manned the flotation machines. When beginning this research, we did not anticpate the results we observed regarding the antiquity of animal tending. We are aware that this study could have cultural significance for the people of Syria. This will be explored in a synthetic report at a later date.

If your manuscript includes photographs of human remains please indicate whether authors obtained permission from descendants or affiliated cultural communities to do so.

Not applicable.

REPUBLIQUE ARABE SYRIENNE

MINISTERE DE LA CULTURE ET DE

L'ORIENTATION NATIONALE

DIRECTION GENERALE DES ANTIQUITES

ET DES MUSEES

Mr. Moore

PERMIS OFFICIEL DE FOUILLES ARCHEOLOGIQUES DANS UN SITE NEOLITHIQUE PRES D'ABU HUREIRA DANS LA HAUTE VALLEE DE L'EUPHRATE

VU la demande présentée le I7. 3. 1972 au nom de l'Université d'Oxford (Musée Pitt Rivers) par Monsieur Andrew Michael T. MOORE, de nationalité Britannique

VU l'étude de cette demande éffectuée par la Direction Générale des Antiquités et des Musées de la République Arabe Syrienne qui a constaté qu'elle est faite conformément aux Articles du Décret Législatif N° 222 du 26/IO/I963 du point de vue juridique.

VU l'approbation du Conseil d'Administration de la Direction Générale des Antiquités et des Musées de la demande en question lors de sa session tenue le 29/4/I972.

Prenant en considération la compétehce de Mr. A. M. T. MOORE et de la capacité scientifique de l'institution qu'il représente :

Nous octroyons ce permis officiel de fouilles archéologiques dans un site Neolithique près d'Abu Hureira dans la haute Vallée de l'Euphrate sous la direction de Mr. Andrew Michael T. MOORE , conformément aux clauses suivantes

I. Mr. A. M. T. MOORE ou son éventuel remplaçant devrait aviser avant deux mois du commencement de chaque saison des fouilles, la Direction Générale des Antiquités et des Musées de toutes modifications éventuelles qu'il pourrait envisager pour la formation de sa mission.

2. Il devrait se conformer strictement aux Articles du Chapitre IV des fouilles Art. 4I-55, du Décret Législatif N° 222 du 26/ IO/ I963 .

3. Il devrait se faire accompagner aux frais de sa mission par un représentant de la Direction Générale des Antiquités et des Musées. Ce représentant se collaborera avec la mission, veillera sur les travaux des fouilles, s'assurera de l'enregistrement des découvertes et servira d'intermédiaire entre la mission et les autorités compétentes.

4. Il devrait livrer à la Direction Générale des Antiquités et des Musées cinq copies de chaque article ou livre publié par lui-même ou par les membres de sa mission sur les travaux de fouilles.

5. Il devrait répendre pour les membres de sa mission vis-À-vis des autorités officielles de la République Arabe Syrienne, et accepter avec les membres de sa mission les mesures de sécurité qui pourraient être prises par les autorités du pays, notamment le Code du Travail, respecter les traditions du pays et ne s'adonner à aucune activité en dehors de son travail archéologique.

6. La mission bénéficiera des clauses du Décret Législatif N° 295 du 2/ I2/ I965 concernant les missions archéologiques étrangères, autorisés à fouiller dans la Région du Barrage de l'Euphrate.

Damas le II/5/I972

Directeur Général
des Antiquités et des Musées

Ministre de la Culture,
et de l'Orientation Nationale

**Syrian Arab Republic**

**Ministry of Culture and the National orientation direction general of Antiquities and Museums.**

**Official permit for archaeological excavations in a Neolithic site near Abu Hureira in the Upper Euphrates Valley**

GIVEN the request presented on 17. 3. 1972 on behalf of Oxford University (Pitt Rivers Museum) by Andrew Michael T. Moore, of British nationality

CONSIDERING the study of this request carried out by the General Directorate of Antiquities and the Museums of the Syrian Arab Republic, which has noted that it has been made, in accordance with the Articles of Legislative Decree Number 222 of 26/10/196 as a point of law.

WITH REGARDS to the approval of the Board of Directors of the Director General of Antiquities and Museums of the request in question during the session held on 29/4/1972.

Taking into consideration the competence of Mr. A.M.T. Moore and the scientific capacity of the institution he represents:

We grant this official permit for archaeological excavations at a Neolithic site near Abu Hureira in the Upper Euphrates Valley under the direction of Mr. Andrew Michael T. Moore, in accordance with the following clauses:

1. Mr. A. M. T. Moore, or his eventual replacement, should notify the General Directorate of Antiquities and Museums two months before the start of each excavation season of any modifications he might consider for the formation of his mission.
2. He should strictly comply with Articles 41–55 within Chapter IV for excavations, of Legislative Decree N. 222 of 26/10/1963.
3. He should be accompanied, at the expense of his mission, by a representative of the General Directorate of Antiquities and Museums. This representative will collaborate with the mission, will watch over the excavation work, will ensure the recording of discoveries and will serve as an intermediary between the mission and the competent authorities.
4. He should deliver to the Directorate General of Antiquities and Museums five copies of each article or book published by himself or by members of his mission on the excavation work.
5. He should represent the members of his mission vis-à-vis the official authorities of the Syrian Arab Republic, and accept (with the members of his mission) the security measures that could be taken by the authorities of the country (in particular the Labor Code), respect the traditions of the country, and not engage in any activity outside of their archaeological work.
6. The mission will benefit from the provisions of Legislative Decree No. 295 of 2/12/1965 concerning foreign archaeological missions, authorized to excavate in the Euphrates Dam Region.

Damascus 11/5/1972
Director General, Ministry of Culture

**DECRET LEGISLATIF N° 295**

Le Chef de l'Etat,

Vu les dispositions de la Constitution provisoire, et l'arrêté du Conseil des Ministres N° 295 du 1.12.1969

**Décrète**

*art. 1 — En dérogation aux dispositions de l'art. 52 du Décret Législatif N° 222 du 26.10.1963, les missions de fouilles étrangères qui seront autorisées à travailler sur les sites archéologiques dont la submersion est prévue par les eaux du barrage de l'Euphrate, pourraient se voir attribuer, à la mise en vigueur du présent décret, la moitié des antiquités mobiles découvertes.*

*art. 2 — Ces missions devront se conformer aux règlements et conditions fixés par la loi sur les antiquités ( décret législatif N° 222 précité ).*

*art. 3 — L'attribution ne s'étend ni aux pièces antiques uniques dans leur genre, ni à celles pouvant compléter des collections importantes illustrant la civilisation de la vallée de l'Euphrate ou celle de la République Arabe Syrienne. Ces antiquités devront prendre place dans les Musées de la République Arabe Syrienne.*

*art. 4 — La quote-part des antiquités dévolues devra être, dans un délai d'un an à compter de sa sortie du territoire arabe syrien, exposée au public dans les musées ou établissements scientifiques du pays dont dépend la mission. Celle-ci devra s'engager par écrit à respecter cette obligation.*

*art. 5 — Les dispositions du présent décret ne s'étendent pas à d'autres sites que pourrait choisir la mission pour compléter ses recherches ou ses études.*

*art. 6 — Les modalités d'attribution des antiquités conformément aux dispositions de l'art. 1 du présent décret législatif feront l'objet d'un arrêté qui sera pris par le Ministre de la Culture, du Tourisme et de l'Orientation Nationale.*

*art. 7 — Le présent décret législatif sera publié, et prendra effet à la date de sa promulgation.*

*Damas le 23.9.1389 H./2.12.1969*

**Le Chef de l'Etat**

**Dr. Noureddine ATASSI**

LEGISLATIVE DECREE No. 295
The head of state,
Having regard to the provisions of the Provisional Constitution, and Order of the Council of Ministers No. 295 of 1.12.1969
Decreed

art. 1 - In accordance with the provisions of art. 52 of Legislative Decree No. 222 of 26.10.1963, the missions of foreign excavations which will be authorized to work on the archaeological sites whose submersion is planned by the waters of the Euphrates dam, could be to have half of the mobile antiquities discovered attributed to the enforcement of this decree.

art. 2 - These missions must comply with the rules and conditions fixed by the law on antiquities (Legislative Decree No. 222 cited above).

art. 3 - The attribution does not extend to antique pieces that are unique in their kind, nor to those that can complete important collections illustrating the civilization of the Euphrates valley or that of the Syrian Arab Republic. These antiquities will have to be placed in the Museums of the Syrian Arab Republic.

art. 4 - The share of devalued antiquities must be, within a period of one year from its release of the Syrian Arab territory, exhibited to the public in museums or scientific establishments of the country on which the mission depends. The latter must undertake in writing to respect this obligation.

art. 5 - The provisions of this decree do not extend to other sites that could be chosen by the mission to complete his research OR his studies.

art. 6 - The methods of allocation of antiquities in accordance with the provisions of art. 1 of the present legislative decree will be the subject of an order which will be taken by the Minister of Culture, Tourism and National Orientation.

Art 7. This Legislative Decree shall be published, and take effect upon the date of its promulgation.

Damascus on 23.9.1389 H./2.12.1969
The head of state
Dr. Noureddine ATASSI
